# Multi-Omics Reveal Additive Cytotoxicity Effects of Aflatoxin B1 and Aflatoxin M1 toward Intestinal NCM460 Cells

**DOI:** 10.3390/toxins14060368

**Published:** 2022-05-25

**Authors:** Ya-Nan Gao, Xue Yang, Jia-Qi Wang, Hui-Min Liu, Nan Zheng

**Affiliations:** 1 Key Laboratory of Quality & Safety Control for Milk and Dairy Products of Ministry of Agriculture and Rural Affairs, Institute of Animal Sciences, Chinese Academy of Agricultural Sciences, Beijing 100193, China; gyn758521@126.com (Y.-N.G.); yangxue234723@126.com (X.Y.); jiaqiwang@vip.163.com (J.-Q.W.); liuhuimin02@caas.cn (H.-M.L.); 2 Laboratory of Quality and Safety Risk Assessment for Dairy Products of Ministry of Agriculture and Rural Affairs, Institute of Animal Sciences, Chinese Academy of Agricultural Sciences, Beijing 100193, China; 3 Milk and Milk Products Inspection Center of Ministry of Agriculture and Rural Affairs, Institute of Animal Sciences, Chinese Academy of Agricultural Sciences, Beijing 100193, China; 4 State Key Laboratory of Animal Nutrition, Institute of Animal Sciences, Chinese Academy of Agricultural Sciences, Beijing 100193, China

**Keywords:** aflatoxin B1, aflatoxin M1, additive effects, multi-omics analyses

## Abstract

Aflatoxin B1 (AFB1) is a common crop contaminant, while aflatoxin M1 (AFM1) is implicated in milk safety. Humans are likely to be simultaneously exposed to AFB1 and AFM1; however, studies on the combined interactive effects of AFB1 and AFM1 are lacking. To fill this knowledge gap, transcriptomic, proteomic, and microRNA (miRNA)-sequencing approaches were used to investigate the toxic mechanisms underpinning combined AFB1 and AFM1 actions in vitro. Exposure to AFB1 (1.25–20 μM) and AFM1 (5–20 μM) for 48 h significantly decreased cell viability in the intestinal cell line, NCM460. Multi-omics analyses demonstrated that additive toxic effects were induced by combined AFB1 (2.5 μM) and AFM1 (2.5 μM) in NCM460 cells and were associated with p53 signaling pathway, a common pathway enriched by differentially expressed mRNAs/proteins/miRNAs. Specifically, based on p53 signaling, cross-omics showed that AFB1 and AFM1 reduced NCM460 cell viability via the hsa-miR-628-3p- and hsa-miR-217-5p-mediated regulation of cell surface death receptor (FAS), and also the hsa-miR-11-y-mediated regulation of cyclin dependent kinase 2 (CDK2). We provide new insights on biomarkers which reflect the cytotoxic effects of combined AFB1 and AFM1 toxicity.

## 1. Introduction

In the natural environment, different crops are likely to be contaminated by mycotoxins during growth and harvesting, especially during environmental stresses such as flooding and assault by insects [1]. Of the different mycotoxins, aflatoxins (AFs) are serious and harmful molecules. Among AFs, the secondary metabolite produced by *Aspergillus*, aflatoxin B1 (AFB1), has attracted considerable research interest due to its universality and high toxicity. Recent studies have shown that colon inflammation and inhibition of embryonic development were associated with the exposure of AFB1 [2,3]. The International Agency for Research on Cancer (IARC) indicated that AFB1 demonstrated clear carcinogenicity to humans and classified the molecule as a class 1 carcinogen [4]. In addition, it is common to find the occurrence of AFB1 in feeds. The mycotoxins detection for a total of 3507 feeds samples collected from China during 2018–2020 showed that more than 81.9% samples were contaminated by AFB1 with 1.2–27.4 μg/kg [5]. Therefore, various strategies, including physical, chemical, biological, and nutritional regulation approaches, have been developed to control AFB1 in feed [6]. When AFB1 is ingested by livestock, it is detoxified by liver microsome monooxygenase and activated by cytochrome P450, with AFB1 finally hydroxylated and metabolized to aflatoxin M1 (AFM1) [7]. AFM1 is then transferred to the mammary glands, urinary system, and other organs and tissues, and is finally excreted via milk and urine. In 2002, the IARC upgraded AFM1 from a class 2 to a class 1 carcinogen.

A recent study has reviewed that human dietary exposure to AFB1 and AFM1 is mainly via grains, grain-based products, and milk products [8]. While previous studies focused on individual toxic molecules, humans are more likely to be exposed to more than one contaminant in the environment [9]. AFs, especially AFB1 and AFM1, are omnipresent contaminants, so it is important to explore their underlying interactive mechanisms. Combined chemical interactions include synergistic, additive, and antagonistic actions. Of these, additive effects represent fundamental interactions, therefore, it is important to clarify combined AFB1 and AFM1 mechanism from an additive perspective.

In humans, the intestinal tract is the first barrier to resist external pollutants; it performs approximately 70% of immune functions in the body. The intestinal tract is also the first organ to contact and absorb mycotoxins from contaminated food or feed [10,11]. Therefore, an in-depth understanding of the mechanisms underpinning a mycotoxin-compromised intestinal tract is warranted [12]. At the cell and molecular level, gene and protein expression reflect the toxicology mechanisms of such mycotoxins. Using transcriptomic and proteomic analyses, we previously reported that ochratoxin A (OTA) and AFM1 induced intestinal dysfunction [13,14]. In addition, microRNAs (miRNA) have recently gained considerable traction as novel biomarkers of disease; miR-34a had important roles in AFB1-induced hepatotoxicity [15]. Additionally, key genes implicated in AFB1-induced hepatotoxicity were screened using miRNA–mRNA regulatory network analysis [16].

However, miRNA/gene/protein biomarkers reflecting mycotoxin toxicity are rarely reported. Only one study reviewed that a panel of genes/proteins/miRNAs could be used as the targets in the further study of cancer development induced by AFB1 and AFM1 [17]. Additionally, when compared with two commonly used colorectal cancer cell lines (HT-29 and Caco-2), the human normal colon mucosal epithelial cell line, NCM460, generated different functions [18]. In this study, to demonstrate additive-effect mechanisms of combined AFM1 and AFB1, bioinformatics analysis of transcriptomic and proteomic data from NCM460 cells, based on gene/protein interaction network analysis was conducted firstly. This was followed by functional miRNA predictions to identify a panel of targets. These findings provide new insights underpinning intestinal toxicity induced by AFB1 and AFM1.

## 2. Results

### 2.1. NCM460 Cytotoxicity Is Induced by AFB1 and AFM1

**Figure 1:**
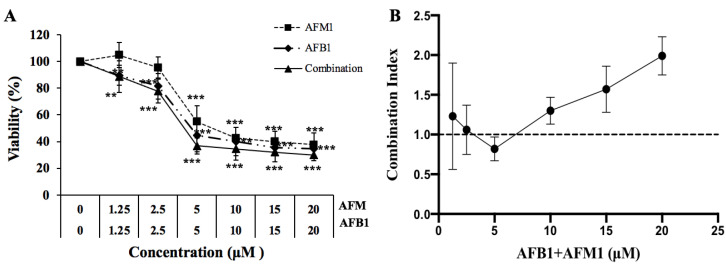
Concentration-response curves and combination index (CI) in NCM460 cells exposed to individual and combined AFM1 and AFB1 at different concentrations for 48 h. (**A**) NCM460 cell viability. Compared with the control group, ** *p* < 0.01, *** *p* < 0.001. (**B**) Combination index (CI) represents the interactive effects of AFM1 and AFB1 at different concentrations. The dotted line indicates additivity, the area under the dotted line synergism, and the area above of the dotted line antagonism. Data are mean values ± standard deviation (SD). Results of 3 independent experiments with 5 replicates. AFM1, aflatoxin M1. AFB1, aflatoxin B1.

To determine the intestinal cytotoxicity induced by AFB1 and AFM1, NCM 460 cells were exposed to different AFB1 and AFM1 concentrations (1.25, 2.5, 5, 10, 15, and 20 μM) either individually or in combination for 48 h. AFB1 significantly decreased (*p* < 0.05) NCM460 cell viability at a low concentration (1.25 μM), while AFM1 caused no effects at the same concentration, indicating that AFB1-induced cytotoxicity was stronger than AFM1 alone (Figure 1A). In addition, half-maximal inhibitory concentration (IC_50_) of 10.47 ± 2.40, 8.10 ± 1.44, and 5.50 ± 1.21 μM, respectively, were determined for AFM1, AFB1, and AFM1 + AFB1 using CalcuSyn software. Therefore, the toxicity order toward NCM460 cells was AFM1 + AFB1 > AFB1 > AFM1.

A combination index (CI) curve of combined AFM1 and AFB1 is shown (Figure 1B). Straight lines in graphs (CI = 1) indicate an additive toxicological effect, with points above (CI > 1) and below (CI < 1) the straight lines indicating antagonistic and synergistic effects, respectively. Exposure to AFM1 + AFB1 in NCM460 cells for 48 h showed an additive effect (CI: 1.06 ± 0.31) at 2.5 μM, a slightly synergistic effect (CI: 0.82 ± 0.15) at 5 μM, and an antagonistic effect (CI: 1.22–2.22) at other concentrations (Figure 1B). Due to the additive effect being the most fundamental type of interaction, we believe that to understand the interaction mechanism of mycotoxins, we must firstly analyze the underlying mechanism of addition Therefore, to investigate the mechanism of combined additive effects from toxins, 2.5 μM AFB1 and 2.5 μM AFM1 were used for omics analysis. Additionally, 2.5 μM was chosen as we were interested in knowing if AFM1, which had no significant effect on cell viability, affected AFB1 toxicity in an additive manner.

### 2.2. Transcriptomic Analysis of NCM460 Cells

**Figure 2:**
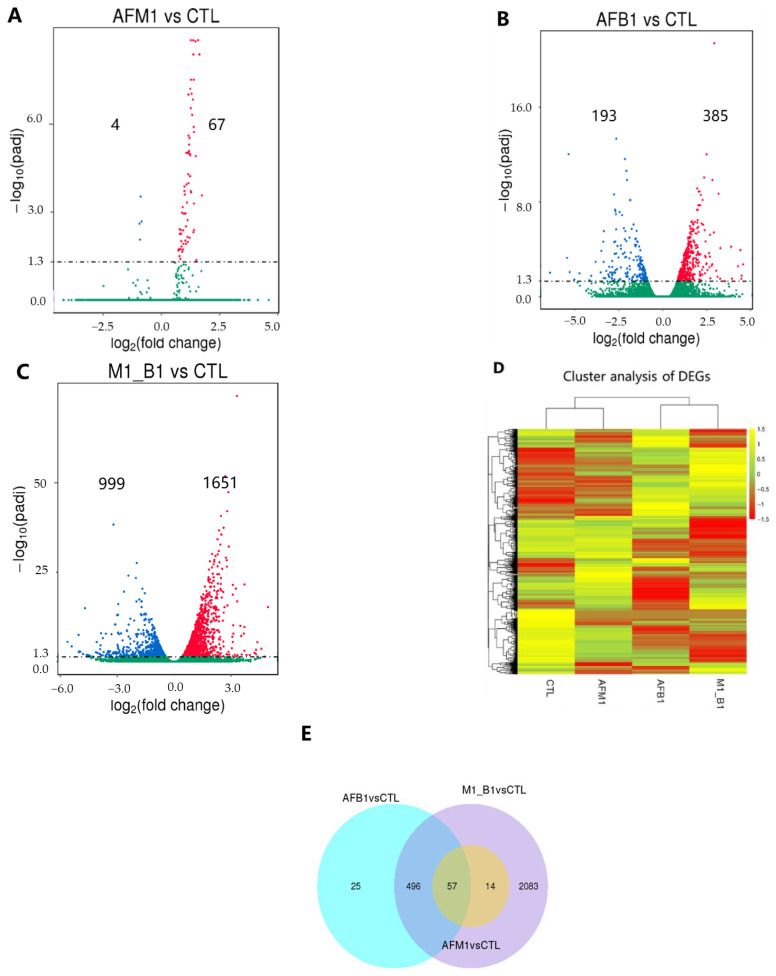
Transcriptome analysis of NCM460 cells induced by individual and combined AFM1 and AFB1 treatments for 48 h. (**A**–**C**) Volcano plots showing the number of differentially expressed genes (DEGs) in AFM1/CTL, AFB1/CTL, and AFM1 + AFB1/CTL groups. (**D**) Hierarchical DEGs clustering in different group in NCM460 cells. (**E**) Venn diagram analysis of AFM1, AFB1, and AFM1 + AFB1 groups. CTL represents the cells with cell medium treatment, AFM1 = 2.5 μM, AFB1 = 2.5 μM, and M1_B1 = AFM1 (2.5 μM) + AFB1 (2.5 μM).

Transcriptome analysis was performed in NCM460 cells exposed to 2.5 μM AFM1, 2.5 μM AFB1, and their combination for 48 h. Volcano plots showed that when compared with control group, the number of differentially expressed genes (DEGs) in AFM1, AFB1, and AFM1 + AFB1 treatments was 71 (67 up and 4 down), 578 (385 up and 193 down), and 2650 (1651 up and 999 down), respectively (Figure 2A–C). Combined toxins generated the most significant changes. A heatmap showed the AFM1 expression profile was similar to the control group, while the AFB1 profile was similar to AFM1 + AFB1 (Figure 2D). This result was consistent with there being no DEGs between AFM1 + AFB1 and AFB1 (Figure S1). A Venn diagram showed that when compared with control group, 2083 unique genes were present in the AFM1 + AFB1 group (Figure 2E).

**Figure 3:**
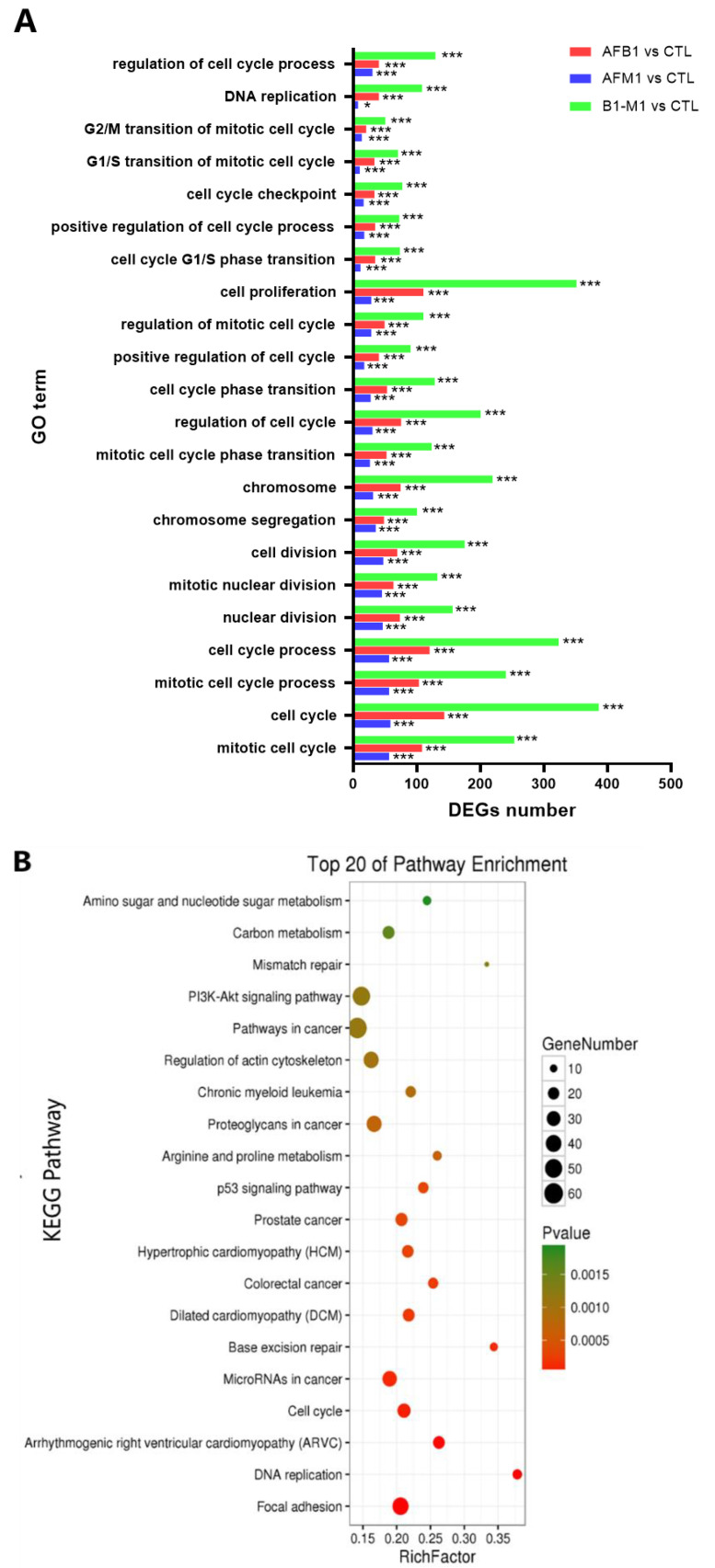

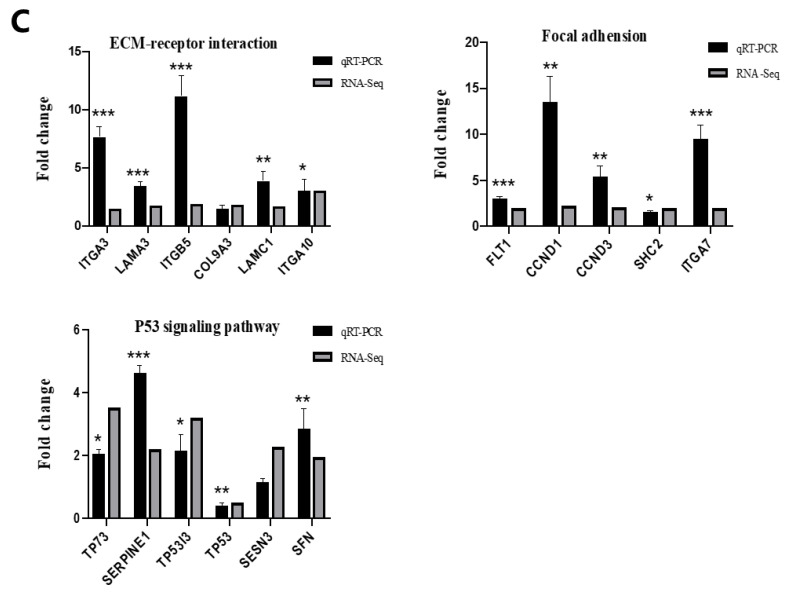
The enrichment and validation of transcriptome. (**A**) Compared with control group, Gene Ontology (GO) terms enriched by the DEGs in AFM1, AFB1, and AFM1 + AFB1, and the significance of GO terms were evaluated by corrected *p* value. (**B**) Kyoto Encyclopedia of Genes and Genomes (KEGG) pathways enriched by 2083 unique genes in the AFM1 + AFB1 treatment group. (**C**) Differentially expressed gene (DEG) in the AFM1 + AFB1/CTL treatment group validation by qRT-PCR. DEGs and their validation by qRT-PCR were analyzed between CTL and AFM1 + AFB1 treatment. Data from qRT-PCR are mean values ± SD. Results of 3 independent experiments with 3 replicates. * *p* < 0.05, ** *p* < 0.01, *** *p* < 0.001.

Subsequently, Gene Ontology (GO) and Kyoto Encyclopedia of Genes and Genomes (KEGG) enrichment analysis was performed. Compared with control group, GO terms were mainly enriched for cell proliferation, cell cycle processes, and cell cycle for individual and combined treatments (Figure 3A). Compared with control group, KEGG analysis enriched by the DEGs in AFM1, AFB1, and AFM1 + AFB1 were shown (Figure S2), which demonstrated that cell cycle and p53 signaling pathway were the most common enrichment pathways in all groups. For KEGG analysis of 2083 unique genes in AFM1 + AFB1 treatment, genes were enriched in focal adhesion, DNA replication, p53 signaling pathway, and the extracellular matrix (ECM) (Figure 3B).

To validate transcriptome data reliability, 17 genes that were canonically related to the intestinal barrier in AFM1 + AFB1 were selected for qRT-PCR. These genes included ECM–receptor interaction (ITGA3, LAMA3, ITGB5, COL9A3, LAMC1, and ITGA10), focal adhesion (FLT1, CCND1, CCND3, SHC2, and ITGA7), and p53 signaling pathway (TP73, SERPINE1, TP53I3, TP53, SESN3, and SFN). Compared with control group, the expression data for these 17 genes in AFM1 + AFB1 treatment were consistent with RNA-sequencing data (Figure 3C).

### 2.3. Proteomic Analysis of NCM460 Cells

**Figure 4:**
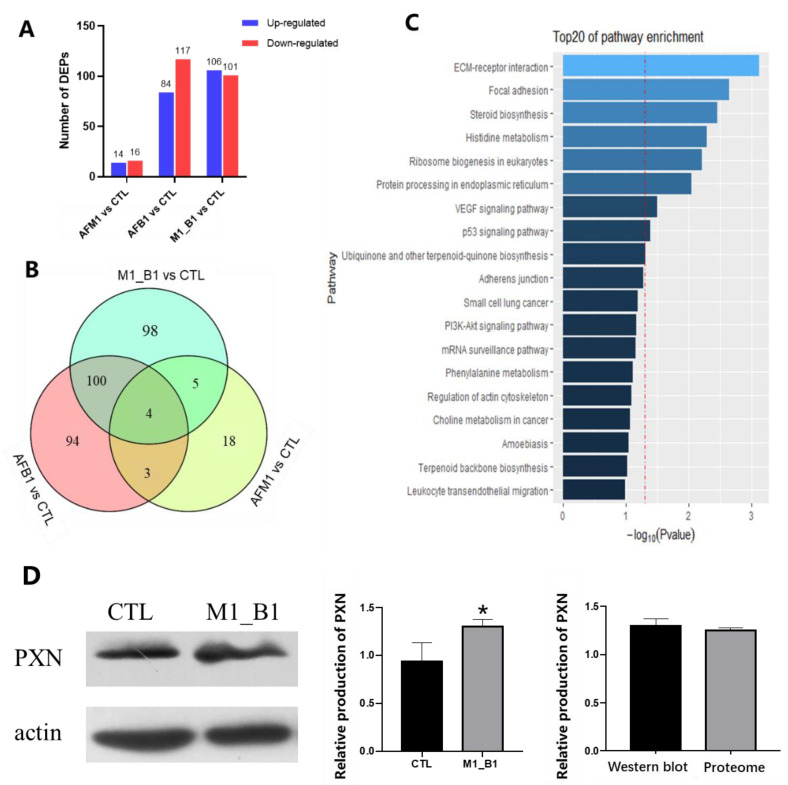
Proteomic analysis of NCM460 cells exposed to individual and combined AFM1 and AFB1 at 2.5 μM for 48 h. To reduce the proteomic sample variability, three replicates of harvested NCM460 cells were pooled into one sample. Each treatment was run in three replicates. (**A**) Differentially expressed proteins (DEPs) in AFM1, AFB1, and AFM1 + AFB1 treatments. (**B**) Venn analysis of AFM1, AFB1, and AFM1 + AFB1 groups. (**C**) Kyoto Encyclopedia of Genes and Genomes (KEGG) analysis of 98 unique DEPs in the AFM1 + AFB1 group. (**D**) Validation of paxillin (PXN) production by western blotting. The results of western blotting are expressed as mean ± SD of three independent experiments. * *p* < 0.05.CTL represents the cells with cell medium treatment, AFM1 = 2.5 μM, AFB1 = 2.5 μM, and M1_B1 = AFM1 (2.5 μM) + AFB1 (2.5 μM).

Proteomic analysis was performed in NCM460 cells exposed to 2.5 μM AFM1, 2.5 μM AFB1, and AFM1 + AFB1 for 48 h. As shown (Figure 4A), when compared with control group, 30 differentially expressed proteins (DEPs) (14 up and 16 down), 201 (84 up and 117 down), and 207 (106 up and 101 down) were observed in AFM1, AFB1, and AFM1 + AFB1 groups, respectively. The number of DEPs among different toxins treatments between AFM1, AFB1, and AFM1 + AFB1 was shown in Figure S3. Venn diagram results showed that when compared with control group, 98 unique DEPs were identified in AFM1 + AFB1 treatment (Figure 4B).

Furthermore, KEGG analysis showed that these 98 unique DEPs were mainly enriched in focal adhesion, ECM receptor interaction, and p53 signaling pathway (Figure 4C). DEPs enrichment in AFM1/CTL, AFB1/CTL, and AFM1 + AFB1/CTL groups showed that p53 signaling pathway and ribosome biogenesis in eukaryotes were key pathways in AFB1-treated cells, while p53 signaling pathway, focal adhesion, and ribosome biogenesis in eukaryotes had vital roles in AFM1 + AFB1-induced cytotoxicity (Table S1). Combining KEGG pathways, p53 signaling pathway had important roles in the intestinal toxicology induced by AFM1 and AFB1.

To confirm our proteomic results, paxillin (PXN) production, which was related to focal adhesion and the ECM pathway, was examined by western blotting. When compared with the control group, AFM1 + AFB1 significantly (*p* < 0.05) increased PXN production. Importantly, western blotting data were consistent with proteome data (Figure 4D).

### 2.4. Cross-Omics Analysis of the Transcriptome and Proteome Induced by AFB1 and AFM1

**Figure 5:**
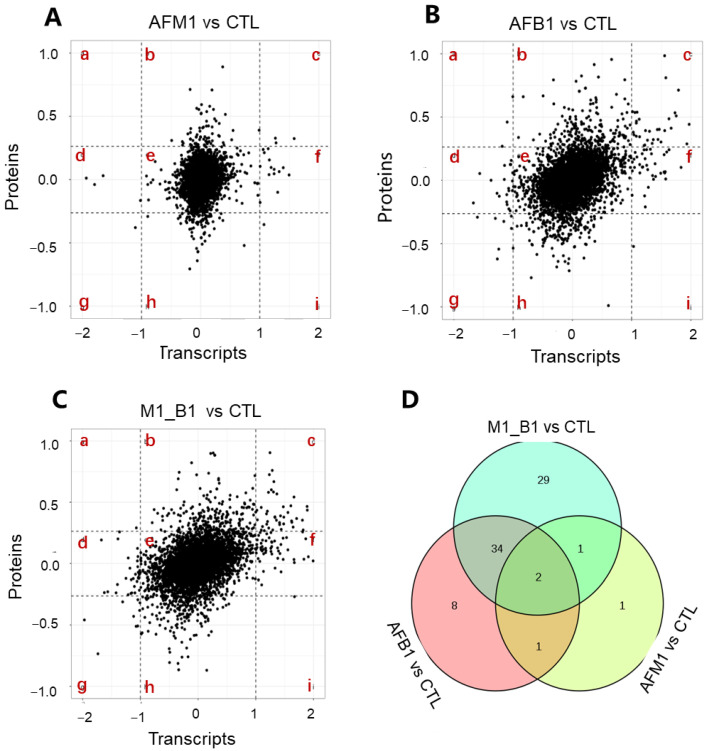

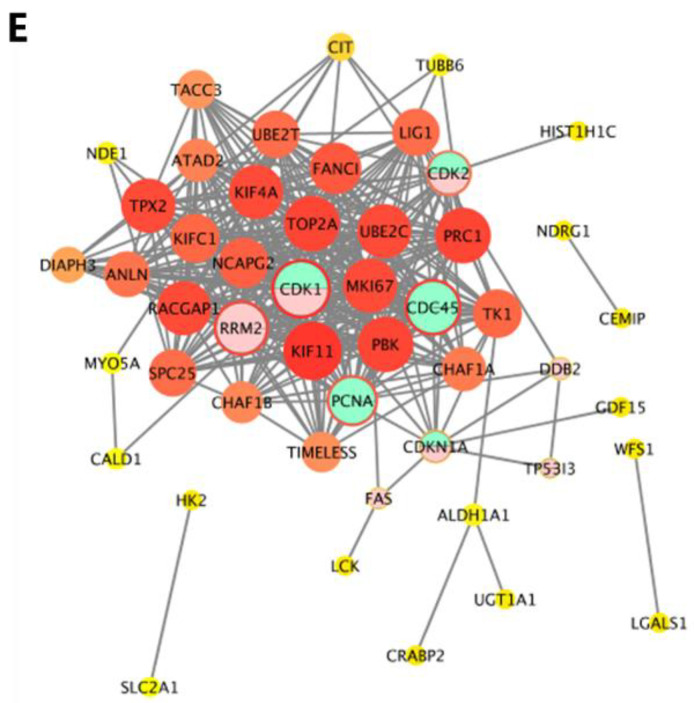
Cross-omics analysis of transcriptomic and proteomic data in individual and combined AFM1 and AFB1 treatment. (**A**–**C**) Comparison of changes in differentially expressed genes (DEGs) and cognate differentially expressed proteins (DEPs) abundance. Quadrant b and h are those correlations that were significant for transcripts only, quadrant d and f are those correlations that were significant for proteins only, quadrant a, c, g and i are those correlations that were significant for both transcripts and proteins, and quadrant e are those that were not significant in either of the two data sets. (**D**) Venn diagram analysis of DEGs and DEPs in quadrants c and g of AFM1, AFB1, and AFM1 + AFB1 groups. (**E**) Protein–protein interaction (PPI) analysis of 49 key proteins related to cell viability in AFM1 + AFB1 group. Different colors represent different pathways. Node size represents connectivity strength. AFM1 = 2.5 μM, AFB1 = 2.5 μM, and M1_B1 = AFM1 (2.5 μM) + AFB1 (2.5 μM).

To narrow down potential targets, a cross-omics analysis of transcriptome and proteome data was conducted. Scatter plot showed the distribution of corresponding proportions of transcripts to proteins (Figure 5). As indicated, most transcript to protein ratios were focused on quadrant e, where both genes and proteins were not differentially expressed. Genes differential expression patterns were consistent with corresponding proteins in quadrant c (unanimous up-regulation) and g (unanimous down-regulation). We observed 5 (3 up and 2 down) proteins associated with DEGs in the AFM1 treatment (Figure 5A). In the AFB1 treatment, 45 (36 up and 9 down) proteins were identified in quadrants c and g (Figure 5B). In the combined treatment, 66 (56 up and 10 down) proteins were associated with DEGs in the transcriptome (Figure 5C). Venn diagram results showed that when compared with the control group, 29 unique proteins were present in the AFM1 + AFB1 group (Figure 5D).

A protein–protein interaction (PPI) network, which included cell viability regulation, was constructed using DEGs and DEPs identified from cross-omics analysis in the AFM1 + AFB1 treatment (Figure 5E). This PPI network was composed of the interactions of 49 nodes and 346 edges, including 17 unique key proteins in the AFM1 + AFB1 group in the Venn diagram. From PPI networks, CDK1 was the central point, with the degree at 30, and played an important role regulating NCM460 cell viability when induced by combined toxins. Additionally, FAS, CDC45, RRM2, PCNA, CDK2, CDKN1A, DDB2, and DIAPH3 were also strongly enriched with cell viability.

### 2.5. miRNA Analysis of NCM460 Cytotoxicity Induced by AFB1 and AFM1

**Figure 6:**
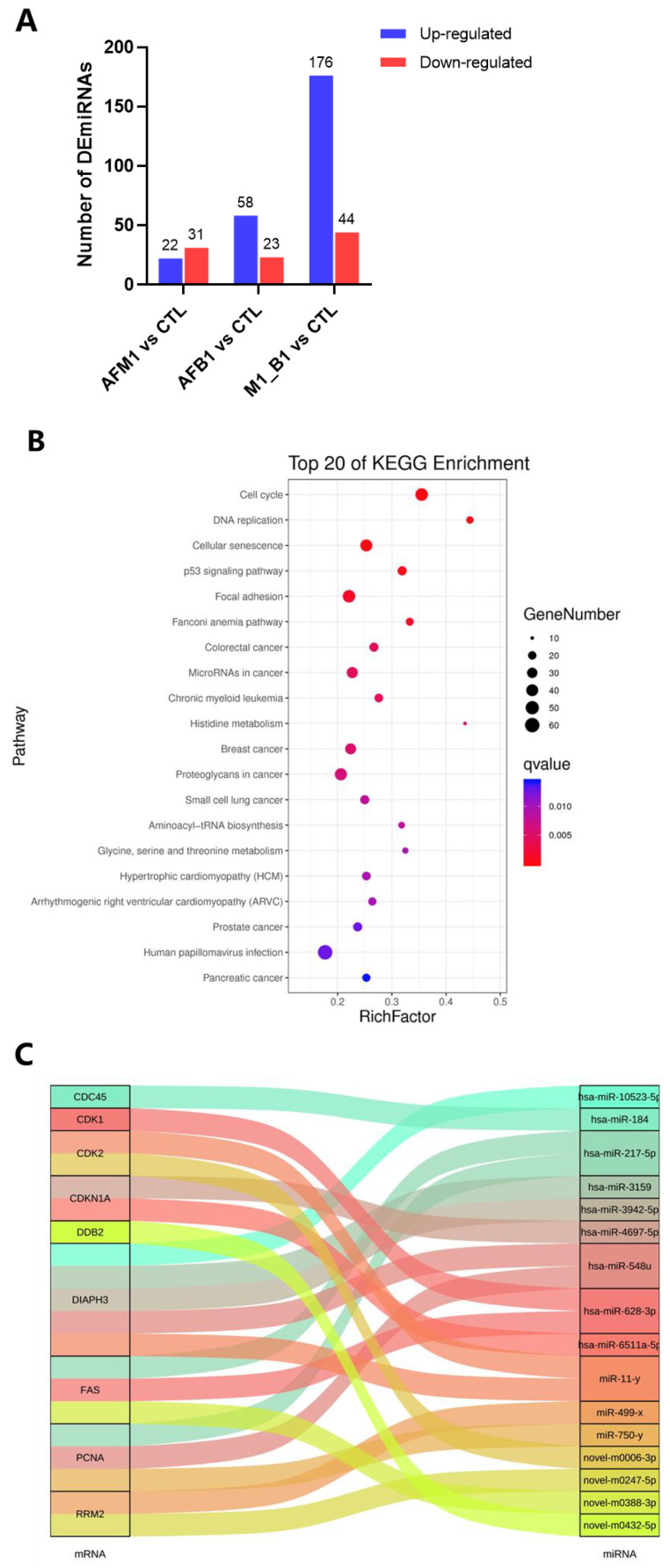
MiRNA analysis in NCM460 cells exposed to individual and combined AFM1 and AFB1 treatments for 48 h. To reduce the miRNA-sequencing sample variability, three replicates of harvested NCM460 cells were pooled into one sample. A total of three samples were prepared from each treatment. (**A**) The number of differentially expressed miRNAs (DEmiRNAs) in AFM1, AFB1, and their combination treatments. (**B**) The top 20 Kyoto Encyclopedia of Genes and Genomes (KEGG) pathways enriched by DEmiRNAs targets. (**C**) A Sankey diagram of the key miRNA–mRNA network related to NCM460 cell viability.

MiRNA sequencing was performed in NCM460 cells exposed to 2.5 μM AFM1, 2.5 μM AFB1, and AFM1 + AFB1 for 48 h. When compared with the control group, 53 (22 up-regulated and 31 down-regulated), 81 (58 up-regulated and 23 down-regulated), and 220 (176 up-regulated and 44 down-regulated) differentially expressed miRNAs (DEmiRNAs) were identified in AFM1, AFB1, and AFM1 + AFB1 groups, respectively (Figure 6A). After determining DEmiRNA target genes, KEGG analysis of target genes in the AFM1 + AFB1 group was performed to predict the functions. Cell viability-related pathways, including the cell cycle, p53 signaling pathway, cellular senescence, and focal adhesion, were significantly enriched after AFM1 + AFB1 treatment (Figure 6B). Combining DEmiRNAs with key DEGs/DEPs targets from PPI analysis, 15 DEmiRNA were putatively identified as key DEmiRNAs related to cell cytotoxicity; these included hsa-miR-628-3p (target CDK1), hsa-miR-217-5p, hsa-miR-628-3p, novel-m0388-3p (target FAS), hsa-miR-184 (target CDC45), hsa-miR-217-5p, hsa-miR-548u, miR-750-y (target PCNA), miR-499-x, novel-m0247-5p (target RRM2), hsa-miR-4697-5p, hsa-miR-6511a-5p (target CDKN1A), novel-m0432-5p (target DDB2), miR-11-y, novel-m0006-3p (target CDK2), hsa-miR-10523-5p, miR-11-y, hsa-miR-3942-5p, and hsa-miR-548u (target DIAPH3 (Figure 6C). Detailed expression levels of these DEmiRNAs are shown (Table S2). Importantly, hsa-miR-628-3p, hsa-miR-548u, hsa-miR-217-5p, and miR-11-y had key roles as they correspond to two kinds of mRNA.

## 3. Discussion

AFs are fungal metabolites in food and feed, with humans likely to be simultaneously exposed to both AFB1 and AFM1. In recent years, the adverse effects of AFB1 and AFM1 on the intestinal barrier have attracted considerable research attention. However, the underlying mechanisms of interactions between toxins are lacking. Therefore, we used multi-omics (transcriptome, proteome, and miRNA-sequencing approaches) to clarify such mechanisms in NCM460 cells—an intestinal epithelial cell line.

AFM1 and AFB1 decreased NCM460 cell viability, with AFB1 toxicity being higher than AFM1 (Figure 1). These results were consistent with our previous study showing that AFB1 cytotoxicity was higher than AFM1 in differentiated and undifferentiated Caco-2 cells [19]. Furthermore, we clarified the interactive effects of AFB1 and AFM1 on cell viability via isobologram analysis (Figure 1), and the interaction of toxins including AFM1, OTA, and zearalenone (ZEN) at different concentrations on cell viability of intestine in vitro were also revealed in our previous study [20]. As the study reported, the type of interaction depends on the concentrations of mycotoxins [21].

From transcriptome, proteome, and miRNA sequencing results, DEGs, DEPs, and DEmiRNAs in combined mycotoxin treatment were higher than in individual mycotoxin treatments (Figure 2A, Figure 4A and Figure 6A). Thus, AFM1 + AFB1 induced more severe intestinal toxicity, via additive effects. Additionally, KEGG pathway analysis of DEGs, DEPs, and DEmiRNAs from AFM1 and AFB1 treatments showed that these toxins affected NCM460 cell viability mainly through p53 signaling pathway. Previously, it was reported that cell viability reduction, induced by various chemical compounds, was directly correlated with p53 induction, and the induction peak appeared at the chemical concentration that caused cell viability to be lower than 80% [22]. In our study, a peak of p53 induction may have occurred due to the fact that 2.5 μM AFB1 and AFM1 maintained the cell viability below 80%. P53 regulates different signaling pathways to prevent cell damage or deterioration. Various cellular stresses could activate p53 signaling pathway [23], which then undergoes post-translational modifications [24] and trans-activates several genes required for cell growth inhibition. P53 activation also results in cell cycle arrest and is associated with the down regulation of cell cycle-related genes/proteins, including CDKN1A, CDK1, and CDC25 [25]. P53 pathway activation also has important roles in the transcriptional regulation of networks responding to cell apoptosis and cell proliferation [26]. The pathway generally responds to mycotoxins, including AFs and OTA. Previously, it was reported that AFB1 caused a G-to-T transversion mutation, which was related to a high frequency of p53 mutation, therefore, the majority of AFB1-associated hepatocellular cancer cases were shown to carry TP53 mutant DNA [27]. P53/p21-mediated cell cycle control was involved in OTA-induced carcinogenic effects [28].

We also screened for key regulators involved in reduced cell viability induced by AFB1 and AFM1. A cross-omics approach using transcriptomics and proteomics was performed and 43 key proteins were selected (Figure 5). Among these regulators, CDK1, CDK2, CDKN1A, DDB2, RRM2, TP53I3, PCNA, and FAS were related to p53 signaling pathway. CDKs are vital cell cycle regulators [29]. AFB1 blocks the cell cycle in S phase by activating p53 [30]. CCND1 overexpression imbalances CDK activity and induces out-of-control cell growth [31]. A previous study showed that AFM1 induced intestinal toxicity by cell cycle arrest in Caco-2 cells via changes in CDK1, SOS1/AKT, and AMPK signaling molecules [32]. ZEN arrested cell cycle progression at the G2/M phase by altering CDK1, CCNB1, CDC25A, and CDC25C expression in porcine granulosa cells [33]. CDK inhibitor p21 (CDKN1A) was involved in regulating the cell process, with p21 knockdown leading to the decrease in cell survival [34]. CDC2/CCNB1 complex inhibition was also involved in citrinin-induced G2/M phase arrest in HEK293 cells [35]. Additionally, in this study, the expression of pro-arrest CDKN1A was increased after AFB1 and AFM1 treatment, consistent with previous data [36]. Additionally, RRM2 was reported as closely linked with cell survival [37,38].

Growing evidence suggests that miRNAs fine-tune gene expression based on different external stimuli, such as mycotoxins [39]. MiRNAs appear to simultaneously interact with various mRNAs, which are involved in key signaling pathways, such as cell processing [40]. For clarification, we jointly analyzed DEmiRNAs with DEGs in combination with DEPs and identified 15 key DEmiRNAs implicated in the regulation of NCM460 cell viability, including hsa-miR-628-3p, hsa-miR-217-5p, and hsa-miR-184 (Figure 6C). These miRNAs have been reported to perform different functions. A new finding suggested that plasma miR-628-3p level may represent a marker to predict the presence of severe atopic keratoconjunctivitis in atopic dermatitis patients [41]. Although miR-628-3p is not yet implicated in intestinal function, we hypothesize it could regulate CDK1 expression. MiR-217-5p could induce apoptosis in colorectal cancer cells by regulating multiple target genes, including PRKCI, BAG3, ITGAV, and MAPK1 in the ERK-MAPK signaling pathway [42]. Similarly, miRNA-217-5p may function as a therapeutic intervention against inflammatory bowel disease [43]. MiR-548u was identified in stored human leukocyte-depleted platelets [44]. In addition, cell function assay and western blotting showed that miR-184 was critical for hepatocellular carcinoma cell proliferation, migration, apoptosis, and autophagy [45].

**Figure 7:**
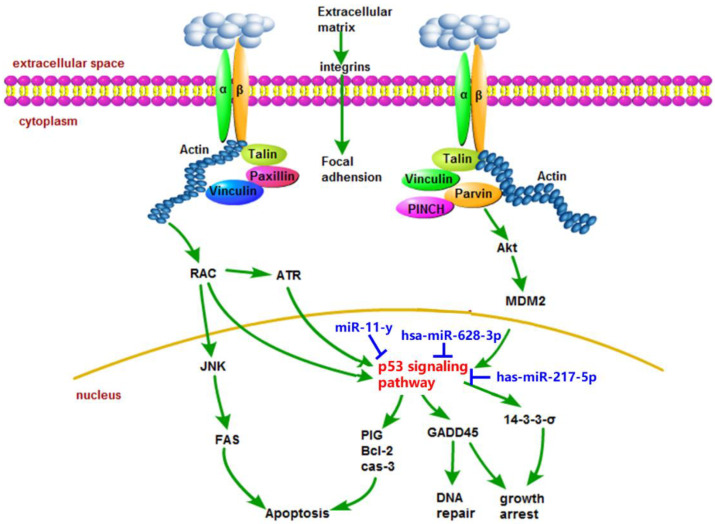
Summary of key miRNA/genes/proteins involved in NCM460 cytotoxicity induced by AFM1 + AFB1 treatment based on multi-omics profiling. Black represents differentially expressed genes (DEGs) and differentially expressed proteins (DEPs). Blue represents differentially expressed miRNAs (DEmiRNAs).

In conclusion, for the first time, we showed that AFM1 and AFB1 exerted additive effects toward NCM460 cell viability via p53 signaling pathway, which appeared to induce changes in downstream regulators, including apoptosis, cell cycle arrest, and DNA repair genes/proteins. Based on p53 signaling pathway, we co-analyzed DEmiRNAs, DEmRNAs, and DEPs, and found that AFM1 + AFB1 reduced NCM460 cell viability via hsa-miR-628-3p and hsa-miR-217-5p FAS regulation, and CDK2 regulation via hsa-miR-11-y (Figure 7). These findings provide a theoretical molecular basis for future mycotoxin risk assessments. Further research is required to comprehensively explore the additive effects of these mycotoxins in the human food chain, and to accurately reflect the potential risk to human intestinal function.

## 4. Materials and Methods

### 4.1. Chemicals

AFB1 and AFM1 reagents (powder) were obtained from Prob Pribolab (Qingdao, China). A 1000 μg/mL stock solution was generated in methanol and stored at −20 °C. Working dilutions (1.25, 2.5, 5, 10, 15, and 20 μM) were prepared in serum-free medium, with the final methanol concentration below 1% (*v*/*v*). Roswell Park Memorial Institute (RPMI) 1640 medium was purchased from Gibco (Grand Island, NY, USA), while fetal bovine serum (FBS), nonessential amino acids (NEAA) and antibiotics (100 units/mL penicillin and 100 mg/mL streptomycin) were purchased from Life Technologies (Carlsbad, CA, USA). Primary and secondary antibody dilution buffer, blocking buffer, 3-(4,5-dimethylthiazol-2-yl)-2,5-diphenyl-2H-tetrazolium bromide (MTT) assay kit, and antifade mounting medium were all obtained from Beyotime Biotechnology (Shanghai, China). Rabbit anti-paxillin (PXN) was purchase from Ab-Mart (CAS: T55274, Shanghai, China). Rabbit anti-β-actin (CAS: bs-10966R), and mouse anti-rabbit IgG (CAS: bs-0295M) were purchased from Bioss (Beijing, China).

### 4.2. Cell Culture and Treatment

NCM460 cells from the American Type Culture Collection (Manassas, VA, USA) were cultured in RPMI 1640 medium supplemented with 4.5 g/L glucose, antibiotics, 1% NEAA, and 10% FBS. Cells were cultured at 37 °C in 5% CO_2_. When the confluence reached approximately 90%, cells were digested with trypsin and then inoculated into 96 well plate with 6 × 10^3^ cells/well for 24 h. Cells were then treated with different AFB1 and AFM1 concentrations (1.25, 2.5, 5, 10, 15, and 20 μM) either individually or in combination for 48 h.

### 4.3. Cytotoxicity Assay

Cell viability of NCM460 cells was examined using MTT assay. After 48 h of individual and combined AFB1 and AFM1 treatments (1.25, 2.5, 5, 10, 15, and 20 μM) in 96-well plates, supernatants were discarded and 0.5 mg/mL 100 μL MTT solution added to wells and plates incubated for 4 h. This solution was removed and 100 μL dimethyl sulfoxide added to dissolve methylazan crystals. The plate was gently shaken for 10 min to completely dissolve crystals, after which absorbance was recorded on a spectrophotometer (Thermo Company, Waltham, MA, USA) at 570 nm and 630 nm. The cell viability was calculated as follows:

Cell viability (%) = (treatment group at 570 nm − treatment group at 630 nm)/(control group at 570 nm − control group at 630 nm) × 100

### 4.4. Isobologram Analysis

CI values were calculated using isobologram analysis according to a formula in our previous study [20]. The interaction type between mixed mycotoxins was quantitatively analyzed by the CI index; CI = 1, <1, and >1 indicated additivity, synergy, and antagonism actions, respectively. Isobologram analysis was performed using CalcuSyn software (Biosoft, Cambridge, UK).

### 4.5. RNA Sequencing and Validation

The transcriptome of RNA sequencing was conducted according to our previous study [13]. In the current study, DEGs were identified as genes with a fold change (FC) ≥2 and false discovery rate <0.05. KEGG pathways, enriched by these DEGs, were investigated. Biological pathways from GO and KEGG analyses were analyzed in a human genome background. To reduce the sample variability, three replicates of harvested NCM460 cells were pooled into one sample. A total of three samples were prepared from each treatment.

To validate RNA-sequencing reliability, qRT-PCR was performed. The conditions were previously described [14]. Primer sequences are shown (Table S3). The 2^−ΔΔCt^ method was used to calculate relative gene expression, with *GAPDH* used as a reference gene.

### 4.6. Proteome Analysis and Validation

Proteomic analysis was conducted as previously reported [13]. Cell supernatants were enzymatically hydrolyzed using reagents from the iTRAQ kit. 114 was labeled as the CTL group, 115 as the AFM1 group, 116 as the AFB1 group, and 117 as the AFB1 + AFM1 (M1-B1) group. Proteins with *p* ≤ 0.05 and FC > 1.2 values were considered to be DEPs. The KEGG analysis enriched by DEPs also conducted as transcriptome. To reduce the sample variability, three replicates of harvested NCM460 cells were pooled into one sample. Each treatment was run in three replicates.

DEPs were validated by western blotting. Lysed NCM460 cell proteins were blocked and incubated with a PXN primary and secondary antibody. Band densities were analyzed in Image J2 software and normalized to human β-actin.

### 4.7. miRNA Sequencing

MiRNA sequencing was conducted as previously described [46]. After deriving total RNA, rRNA, scRNA, snRNA, snoRNA, and tRNA were identified and removed using GeneBank (Release 209.0) and Rfam databases (Release 11.0). The criteria for identifying DEmiRNAs were *p* < 0.05 and FC ≥ 2 values. Target mRNAs for DEmiRNAs were predicted by miRanda (Version 3.3a) and TargetScan (Version 7.0) with Spearman Rank Correlation Coefficient (SRCC) ≤ −0.5. The relationship between miRNA–mRNA was illustrated by Sankey plot. KEGG analyses were also performed to functionally annotate target mRNAs. To reduce the miRNA-sequencing sample variability, three replicates of harvested NCM460 cells were pooled into one sample. A total of three samples were prepared from each treatment.

### 4.8. Statistical Analysis

Cell viability data were analyzed in GraphPad Prism 8.0 (San Diego, CA, USA), and one-way analysis of variance and t-test methods were used for statistical analyses. A *p* < 0.05 value was regarded as statistically significant.

## Data Availability

Data are available upon request; please contact the contributing authors.

## Supplementary Materials

The following are available online at https://www.mdpi.com/article/10.3390/toxins14060368/s1, Figure S1: The DEGs of AFB1 and the combination of AFB1 and AFM1 compared with AFM1 group. Figure S2: The overviews of DEGs. The GO (A-C) and KEGG (D-F) enrichment analysis for different treatment group (AFB1, AFM1 and M1_B1 group) compared with CTL group in order. Figure S3: The number of differential expression proteins in AFB1/AFM1, M1_B1/AFM1 and M1_B1/AFB1. Table S1: The significant enriched KEGGs in AFB1/CTL, AFM1/CTL, AFB1/AFM1 and M1_B1/AFM1. Table S2: The DEmiRNAs related with NCM460 cell viability in AFB1 + AFM1 treatment. Table S3: Primer sequences for quantified of genes by qRT-PCR.

## References

[1] Bhat R., Reddy K.R. (2017). Challenges and issues concerning mycotoxins contamination in oil seeds and their edible oils: Updates from last decade. Food Chem..

[2] Dey D.K., Kang S.C. (2020). Aflatoxin B1 induces reactive oxygen species-dependent caspase-mediated apoptosis in normal human cells, inhibits Allium cepa root cell division, and triggers inflammatory response in zebrafish larvae. Sci. Total Environ..

[3] Zhou J., Tang L.L., Wang J.S. (2021). Aflatoxin B1 Induces Gut-Inflammation-Associated Fecal Lipidome Changes in F344 Rats. Toxicol. Sci..

[4] Dey D.K., Chang S.N., Kang S.C. (2021). The inflammation response and risk associated with aflatoxin B1 contamination was minimized by insect peptide CopA3 treatment and act towards the beneficial health outcomes. Environ. Pollut..

[5] Zhao L., Zhang L., Xu Z.J., Liu X.D., Chen L.Y., Dai J.F., Karrow N.A., Sun L.H. (2021). Occurrence of Aflatoxin B1, deoxynivalenol and zearalenone in feeds in China during 2018–2020. J. Anim. Sci. Biotechnol..

[6] Liu M., Zhao L., Gong G.X., Zhang L., Shi L., Dai J.F., Han Y.M., Wu Y.Y., Khalil M.M., Sun L.H. (2022). Invited review: Remediation strategies for mycotoxin control in feed. Anim. Feed. Sci. Technol..

[7] Deng J., Zhao L., Zhang N.Y., Karrow N.A., Krumm C.S., Qi D.S., Sun L.H. (2018). Aflatoxin B1 metabolism: Regulation by phase I and II metabolizing enzymes and chemoprotective agents. Mutat. Res. Rev. Mutat. Res..

[8] Dey D.K., Kang J., Bajpai V.K., Kim K., Lee H., Sonwal S., Simal-Gandara J., Xiao J.B., Ali S., Huh Y.S. (2022). Mycotoxins in food and feed: Toxicity, preventive challenges, and advanced detection techniques for associated diseases. Crit. Rev. Food Sci. Nutr..

[9] Heys K.A., Shore R.F., Pereira M.G., Jones K.C., Martin F.L. (2016). Risk assessment of environmental mixture effects. RSC Advances.

[10] Grenier B., Applegate T.J. (2013). Modulation of intestinal functions following mycotoxin ingestion: Meta-analysis of published experiments in animals. Toxins.

[11] Kowalska K., Habrowska-Gorczynska D.E., Piastowska-Ciesielska A.W. (2016). Zearalenone as an endocrine disruptor in humans. Environ. Toxicol. Pharmacol..

[12] Gao Y., Meng L., Liu H., Wang J., Zheng N. (2020). The Compromised Intestinal Barrier Induced by Mycotoxins. Toxins.

[13] Gao Y., Li S., Bao X., Luo C., Yang H., Wang J., Zhao S., Zheng N. (2018). Transcriptional and Proteomic Analysis Revealed a Synergistic Effect of Aflatoxin M1 and Ochratoxin A Mycotoxins on the Intestinal Epithelial Integrity of Differentiated Human Caco-2 Cells. J. Proteome Res..

[14] Yang X., Gao Y., Yan Q., Bao X., Zhao S., Wang J., Zheng N. (2019). Transcriptome Analysis of Ochratoxin A-Induced Apoptosis in Differentiated Caco-2 Cells. Toxins.

[15] Zhu L., Gao J., Huang K., Luo Y., Zhang B., Xu W. (2015). miR-34a screened by miRNA profiling negatively regulates Wnt/β-catenin signaling pathway in Aflatoxin B1 induced hepatotoxicity. Sci. Rep..

[16] Zhang Z., Tang D., Wang B., Wang Z., Liu M. (2019). Analysis of miRNA-mRNA regulatory network revealed key genes induced by aflatoxin B1 exposure in primary human hepatocytes. Mol. Genet. Genom. Med..

[17] Marchese S., Polo A., Ariano A., Velotto S., Costantini S., Severino L. (2018). Aflatoxin B1 and M1: Biological Properties and Their Involvement in Cancer Development. Toxins.

[18] Long X., Wong C.C., Tong L., Chu E.S.H., Szeto C.H., Go M.Y.Y., Coker O.O., Chan A.W.H., Chan F.K.L., Sung J.J.Y. (2019). Peptostreptococcus anaerobius promotes colorectal carcinogenesis and modulates tumour immunity. Nat. Microbiol..

[19] Zhang J., Zheng N., Liu J., Li F.D., Li S.L., Wang J.Q. (2015). Aflatoxin B1 and aflatoxin M1 induced cytotoxicity and DNA damage in differentiated and undifferentiated Caco-2 cells. Food Chem. Toxicol..

[20] Gao Y., Wang J., Li S., Zhang Y., Zheng N. (2016). Aflatoxin M1 cytotoxicity against human intestinal Caco-2 cells is enhanced in the presence of other mycotoxins. Food Chem. Toxicol..

[21] Clarke R., Connolly L., Frizzell C., Elliott C.T. (2014). Cytotoxic assessment of the regulated, co-existing mycotoxins aflatoxin B1, fumonisin B1 and ochratoxin, in single, binary and tertiary mixtures. Toxicon.

[22] Di Paolo C., Muller Y., Thalmann B., Hollert H., Seiler T.B. (2018). p53 induction and cell viability modulation by genotoxic individual chemicals and mixtures. Environ. Sci. Pollut. Res. Int..

[23] Volarevic S., Stewart M.J., Ledermann B., Zilberman F., Terracciano L., Montini E., Grompe M., Kozma S.C., Thomas G. (2000). Proliferation, but not growth, blocked by conditional deletion of 40S ribosomal protein S6. Science.

[24] Brooks C.L., Gu W. (2003). Ubiquitination, phosphorylation and acetylation: The molecular basis for p53 regulation. Curr. Opin. Cell Biol..

[25] Engeland K. (2018). Cell cycle arrest through indirect transcriptional repression by p53: I have a DREAM. Cell Death Differ..

[26] Kumari R., Kohli S., Das S. (2014). p53 regulation upon genotoxic stress: Intricacies and complexities. Mol. Cell Oncol..

[27] Engin A.B., Engin A. (2019). DNA damage checkpoint response to aflatoxin B1. Environ. Toxicol. Pharmacol..

[28] Kuroda K., Hibi D., Ishii Y., Yokoo Y., Takasu S., Kijima A., Matsushita K., Masumura K., Kodama Y., Yanai T. (2015). Role of p53 in the progression from ochratoxin A-induced DNA damage to gene mutations in the kidneys of mice. Toxicol. Sci..

[29] Bury M., Le Calve B., Lessard F., Dal Maso T., Saliba J., Michiels C., Ferbeyre G., Blank V. (2019). NFE2L3 Controls Colon Cancer Cell Growth through Regulation of DUX4, a CDK1 Inhibitor. Cell Rep..

[30] Kim J., Park S.H., Do K.H., Kim D., Moon Y. (2016). Interference with mutagenic aflatoxin B1-induced checkpoints through antagonistic action of ochratoxin A in intestinal cancer cells: A molecular explanation on potential risk of crosstalk between carcinogens. Oncotarget.

[31] Qie S., Diehl J.A. (2016). Cyclin D1, cancer progression, and opportunities in cancer treatment. J. Mol. Med..

[32] Bao X.Y., Li S.L., Gao Y.N., Wang J.Q., Zheng N. (2019). Transcriptome analysis revealed that aflatoxin M1 could cause cell cycle arrest in differentiated Caco-2 cells. Toxicol. Vitr..

[33] Li N., Liu X.L., Zhang F.L., Tian Y., Zhu M., Meng L.Y., Dyce P.W., Shen W., Li L. (2020). Whole-transcriptome analysis of the toxic effects of zearalenone exposure on ceRNA networks in porcine granulosa cells. Environ. Pollut..

[34] Yosef R., Pilpel N., Papismadov N., Gal H., Ovadya Y., Vadai E., Miller S., Porat Z., Ben-Dor S., Krizhanovsky V. (2017). p21 maintains senescent cell viability under persistent DNA damage response by restraining JNK and caspase signaling. EMBO J..

[35] Chang C.H., Yu F.Y., Wu T.S., Wang L.T., Liu B.H. (2011). Mycotoxin citrinin induced cell cycle G2/M arrest and numerical chromosomal aberration associated with disruption of microtubule formation in human cells. Toxicol. Sci..

[36] Fischer N.W., Prodeus A., Malkin D., Gariepy J. (2016). p53 oligomerization status modulates cell fate decisions between growth, arrest and apoptosis. Cell Cycle.

[37] Giannattasio M., Branzei D. (2017). S-phase checkpoint regulations that preserve replication and chromosome integrity upon dNTP depletion. Cell Mol. Life Sci..

[38] Li J., Pang J., Liu Y., Zhang J., Zhang C., Shen G., Song L. (2018). Suppression of RRM2 inhibits cell proliferation, causes cell cycle arrest and promotes the apoptosis of human neuroblastoma cells and in human neuroblastoma RRM2 is suppressed following chemotherapy. Oncol. Rep..

[39] Bartel D.P. (2004). MicroRNAs: Genomics, Biogenesis, Mechanism, and Function. Cell.

[40] Baek D., Villen J., Shin C., Camargo F.D., Gygi S.P., Bartel D.P. (2008). The impact of microRNAs on protein output. Nature.

[41] Ueta M., Nishigaki H., Komai S., Sotozono C., Kinoshita S. (2021). Difference in the plasma level of miR-628-3p in atopic dermatitis patients with/without atopic keratoconjunctivitis. Immun. Inflamm. Dis..

[42] Flum M., Kleemann M., Schneider H., Weis B., Fischer S., Handrick R., Otte K. (2018). miR-217-5p induces apoptosis by directly targeting PRKCI, BAG3, ITGAV and MAPK1 in colorectal cancer cells. J. Cell Commun. Signal..

[43] Zhang L.C., Wu X.Y., Yang R.B., Chen F., Liu J.H., Hu Y.Y., Wu Z.D., Wang L.F., Sun X. (2021). Recombinant protein Schistosoma japonicum-derived molecule attenuates dextran sulfate sodium-induced colitis by inhibiting miRNA-217-5p to alleviate apoptosis. World J. Gastroenterol..

[44] Dahiya N., Sarachana T., Kulkarni S., Wood W.H., Zhang Y., Becker K.G., Wang B.D., Atreya C.D. (2017). miR-570 interacts with mitochondrial ATPase subunit g (ATP5L) encoding mRNA in stored platelets. Platelets.

[45] Huang W., Huang F., Lei Z., Luo H. (2020). LncRNA SNHG11 Promotes Proliferation, Migration, Apoptosis, and Autophagy by Regulating hsa-miR-184/AGO2 in HCC. OncoTargets Ther..

[46] Yang X., Gao Y., Huang S., Su C., Wang J., Zheng N. (2021). Whole transcriptome-based ceRNA network analysis revealed ochratoxin A-induced compromised intestinal tight junction proteins through WNT/Ca(2+) signaling pathway. Ecotoxicol. Environ. Saf..

