# Supplementary material for: Multi-Omics Reveal Additive Cytotoxicity Effects of Aflatoxin B1 and Aflatoxin M1 toward Intestinal NCM460 Cells
Source: Toxins (Basel). 2022 May 25;14(6):368. doi: 10.3390/toxins14060368 (PMC9231300; doi:10.3390/toxins14060368)
Supplement: Supplementary file 1 [file toxins-14-00368-s001.zip › toxins-1695804-supplementary.pdf]

Supplementary Materials: Multi-Omics Reveal Additive Cytotoxicity Effects of Aflatoxin B1 and Aflatoxin M1 toward Intestinal NCM460 Cells

Ya-Nan Gao, Xue Yang, Jia-Qi Wang, Hui-Min Liu and Nan Zheng

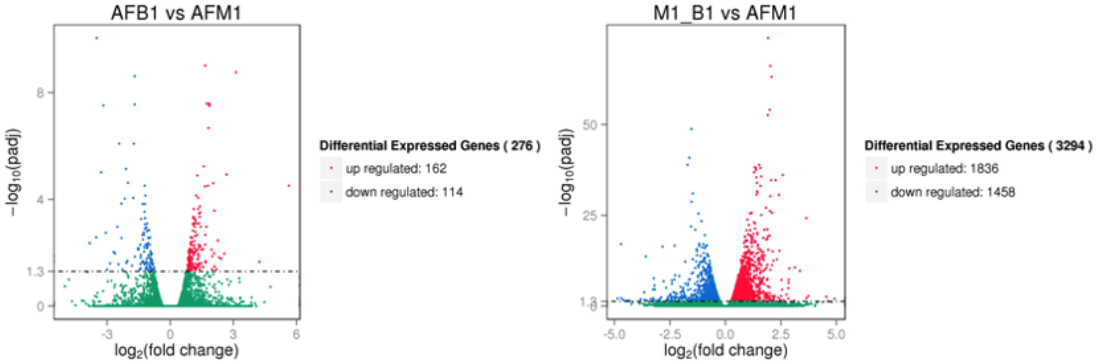

Figure S1. The DEGs of AFB1 and the combination of AFB1 and AFM1 compared with AFM1 group.

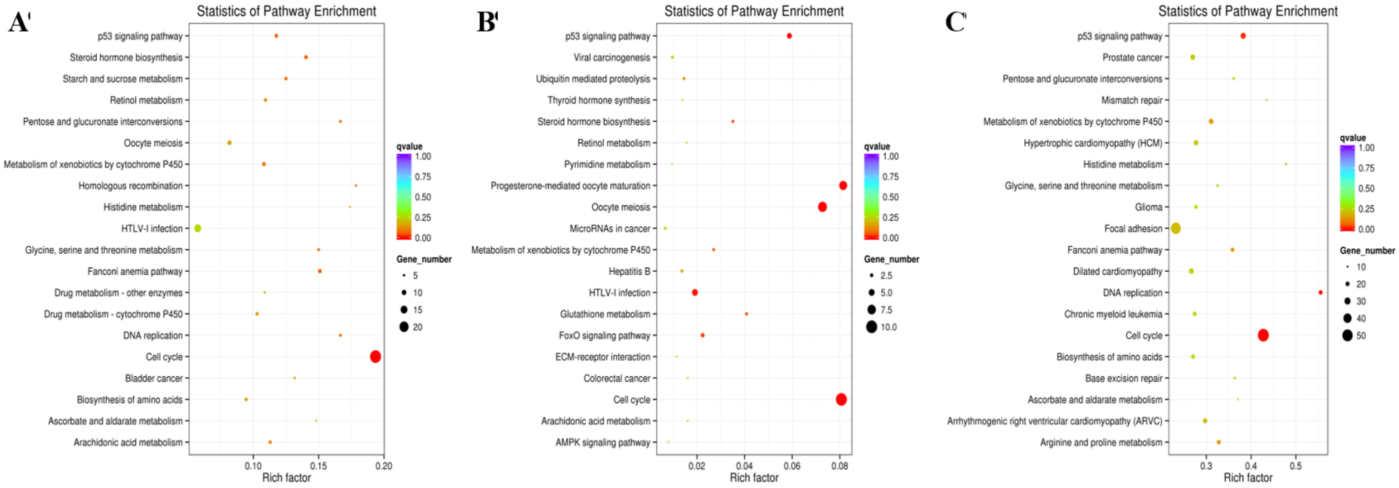

Figure S2. The overviews of DEGs. KEGG (D-F) enrichment analysis for different treatment AFB1 (A), AFM1 (B) and M1_B1 (C) group compared with CTL group in order.

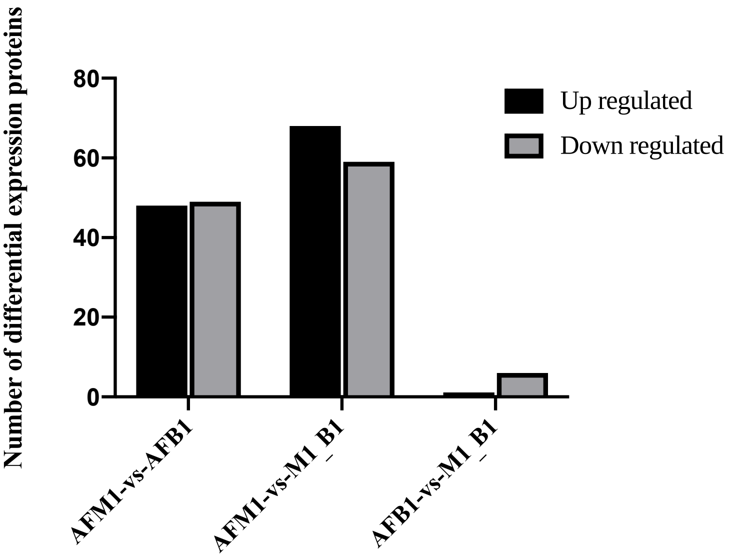

Figure S3. The number of differential expression proteins in AFB1/AFM1, M1_B1/AFM1 and M1_B1/AFB1.

Table S1. The significant enriched KEGGs in AFB1/CTL, AFM1/CTL, AFB1/AFM1 and M1_B1/AFM1.

Group	UP	DOWN
AFB1/CTL	P53 signaling pathway	Ribosome biogenesis in eukaryotes
M1_B1/CTL	P53 signaling pathway<br>Focal adhesion	Ribosome biogenesis in eukaryotes
AFB1/AFM1	Ribosome biogenesis in eukaryotes	/
M1_B1/AFM1	P53 signaling pathway	Ribosome biogenesis in eukaryotes

Table S2. The DEmiRNAs related with NCM460 cell viability in AFB1+AFM1 treatment.

miRNA	Control_tpm	AFB1+AFM1_tpm	log2(fc)	p-values
hsa-miR-184	2.55	0.87	-1.54	0.0001
hsa-miR-628-3p	37.92	16.82	-1.17	8.46E-05
miR-11-y	0.43	0.01	-5.43	0.027
novel-m0006-3p	0.31	0.01	-4.93	0.021
novel-m0432-5p	0.49	0.05	-3.21	0.015
hsa-miR-217-5p	0.82	0.10	-2.96	0.012
novel-m0388-3p	0.37	0.01	-5.19	0.006
miR-750-y	5.45	0.32	-4.07	2.33E-11
hsa-miR-548u	3.02	1.38	-1.13	0.005
miR-499-x	1.60	0.73	-1.14	0.05
novel-m0247-5p	19.65	7.59	-1.37	0.0007
hsa-miR-4697-5p	0.70	0.22	-1.65	0.015
hsa-miR-6511a-5p	0.72	0.05	-3.78	0.001
hsa-miR-10523-5p	0.37	0.06	-2.64	0.05
hsa-miR-3942-5p	2.02	0.72	-1.49	0.02

Table S3. Primer sequences for quantified of genes by qRT-PCR.

Gene name	Forward Primer Sequence (5′–3′)	Reverse Primer Sequence (5′–3′)
COL9A3	GAGACCAGGGTATTGCAGGT	ACTGCCAGACTCTCCTTTGG
ITGA3	TACAGGCGAAACATCACCCT	ATGGAGAAGAAGCCGTGGAA
ITGA10	TAGCTGCTCCTCTCTCCTGA	CTGAGGTCTGGGCTGTGTTA
ITGB5	GTGGAGTTGTCAGTCTGGGA	TGTCCCCAATCTTCAGACCC
LAMA3	TCACACGGGCACTAAGAACT	CGTGTGCCATTTCCCATCAT
LAMC1	GCAGTGACAACATCGATCCC	CGGTCACAATAGAAGCCAGC
FLT1	TTACCGAATGCCACCTCCAT	CTTGGGTTTGCTGTCAGTCC
SHC2	CAGCTTCATCCACAAACCCG	TAGAGCGGAGAACCTCGATG
ITGA7	GATGAGTTGGATGGTGGGGA	AGGTAGTGGCTATCAGGGGA
TP73	GGTGTCCAAACTGCATCGAG	GTACTGCTCGGGGATCTTCA
SERPINE1	CATGTTCATTGCTGCCCCTT	CCTGGTCATGTTGCCTTTCC
CCND1	CAAGCTCAAGTGGAACCTGG	CGTGTTTGCGGATGATCTGT
CCND3	CTCAGGGAAGCCTCTCAGAC	ACAGGTGTATGGCTGTGACA
TP53I3	GAAGAACGTCAACTGCCTGG	GGTGATCAGACTTCCTCGCT
TP53	GCGAGCACTGTCCAACAAC	GAACATCTCGAAGCGCTCA
SESN3	AGGCAGCAACTTTGGGATTG	AGACGCCTCTTCATCTTCCC
SFN	GGGAGAAGGTGGAGACTGAG	GCGGTAGTAGTCACCCTTCA
GAPDH	GAAGGTGAAGGTCGGAGTCAAC	CAGAGTTAAAAGCAGCCCTGGT
